# Chopart dislocations: a review of diagnosis, treatment and outcomes

**DOI:** 10.1007/s00402-023-05040-4

**Published:** 2023-09-15

**Authors:** Tobias S. N. Metcalfe, Junaid Aamir, Lyndon W. Mason

**Affiliations:** 1 Liverpool Orthopaedic and Trauma Service, Liverpool University Hospitals NHS Foundation Trust, Lower Lane, Liverpool, L9 7AL UK; 2 School of Medicine, University of Liverpool, Cedar House, Ashton Street, Liverpool, L69 3GE UK

**Keywords:** Chopart dislocation, Chopart pure-dislocation, Chopart fracture-dislocation, Chopart joint, Midtarsal Joint, Chopart injury, Trauma

## Abstract

**Introduction:**

Chopart injuries can be allocated into 4 broad groups, ligamentous injury with or without dislocation and fracture with or without dislocation, which must occur at the talonavicular joint (TNJ) and/or calcaneocuboid joint (CCJ). Chopart dislocations are comprised of pure-dislocations and fracture-dislocations. We aim to review the literature, to enable evidence-based recommendations.

**Methods:**

A literature search was conducted to identify relevant articles from the electronic databases, PubMed, Medline and Scopus. The PRISMA flow chart was used to scrutinise the search results. Articles were screened by title, abstract and full text to confirm relevance.

**Results:**

We identified 58 papers for analysis, 36 case reports, 4 cohort studies, 4 case series and 14 other articles related to the epidemiology, diagnosis, treatment and outcomes of Chopart dislocations. Diagnostic recommendations included routine imaging to contain computed tomography (CT) and routine examination for compartment syndrome. Treatment recommendations included early anatomical reduction, with restoration and maintenance of column length and joint congruency. For both pure-dislocations and fracture-dislocations urgent open reduction and internal fixation (ORIF) provided the most favourable long-term outcomes.

**Conclusions:**

Chopart dislocations are a complex heterogenous midfoot injury with historically poor outcomes. There is a relative paucity of research discussing these injuries. We have offered evidence-based recommendations related to the clinical and surgical management of these rare pathologies.

## Introduction

The midtarsal or transverse-tarsal joint, is otherwise known eponymously as the Chopart joint, after French surgeon François Chopart described an amputation through the articulation between the hindfoot and the midfoot [1]. The Chopart joint is made up of the TNJ and calcaneocuboid joint CCJ. The TNJ makes up part of the coxa pedis (talocalcaneonavicular joint) which enables pronation and supination of the tarsus [2]. The CCJ provides approximately 25° of rotation for hindfoot eversion and inversion [3]. The Chopart joint enables hindfoot pivot, allowing the forefoot to remain inverted or everted on heel inversion, locking the Chopart joint, stabilizing the midfoot during gait push-off phase [4]. Thus, the Chopart joint is fundamental for normal foot function and requires strong ligamentous support. The TNJ is supported superiorly by the dorsal talonavicular ligament and medial limb of the bifurcate ligament and inferiorly by the spring ligament (calcaenonavicular ligament) comprised of the medioplantar oblique, inferoplantar longitudinal and superomedial components [4]. The CCJ is supported superiorly by the dorsal calcaneocuboid ligament and the lateral limb of the bifurcate ligament and inferiorly by the short plantar ligament (plantar calcaneocuboid ligament) [4].

**Fig. 1:**
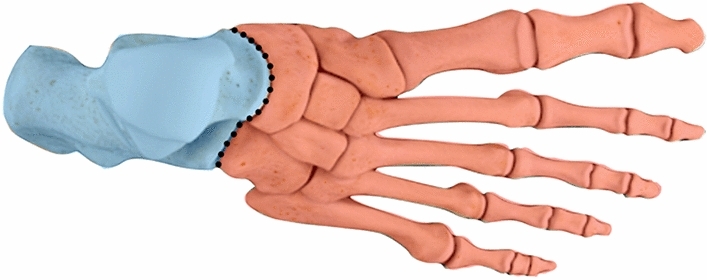
Dorsoplantar Illustration of the Chopart joint and cyma line dividing the midfoot and hindfoot. This congruent S-shaped line corresponds to the Chopart joint

The cyma line (Fig. 1) represents the Chopart joint and can be observed via dorsoplantar and lateral views [5], where radiological discrepancy indicates pathology. Therefore, Initial imaging should start with radiographs in 3 views dorsoplantar, lateral and oblique [6]. Oblique views are optimal for visualising fractures of the anterior calcaneal process [4].

**Fig. 2:**
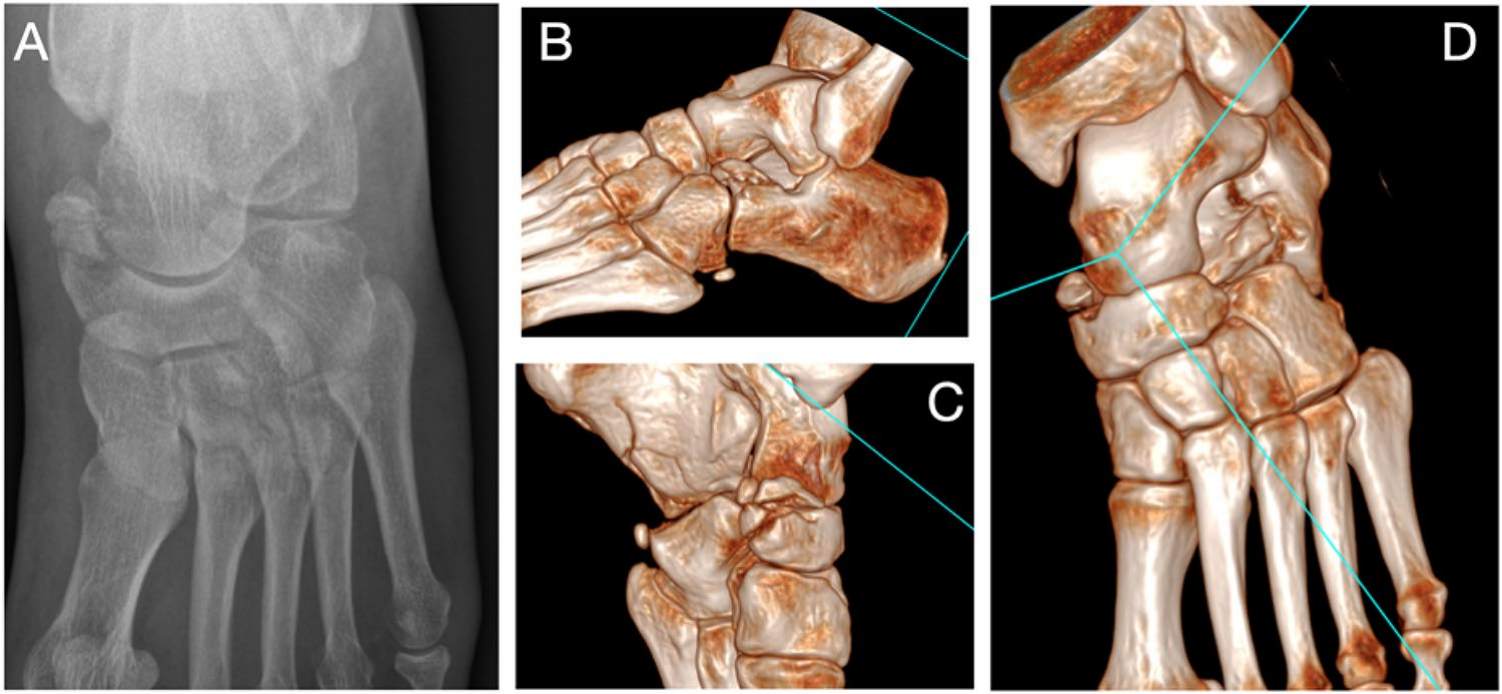
Chopart fracture without dislocation example. Anteroposterior radiograph (**A**) and 3D Surface rendering CT (**B**, **C** and **D**) of a Lateral compression (Anterior process calcaneum) Chopart joint injury and medial distraction injury with tibialis posterior and spring ligament avulsion from the navicular

**Fig. 3:**
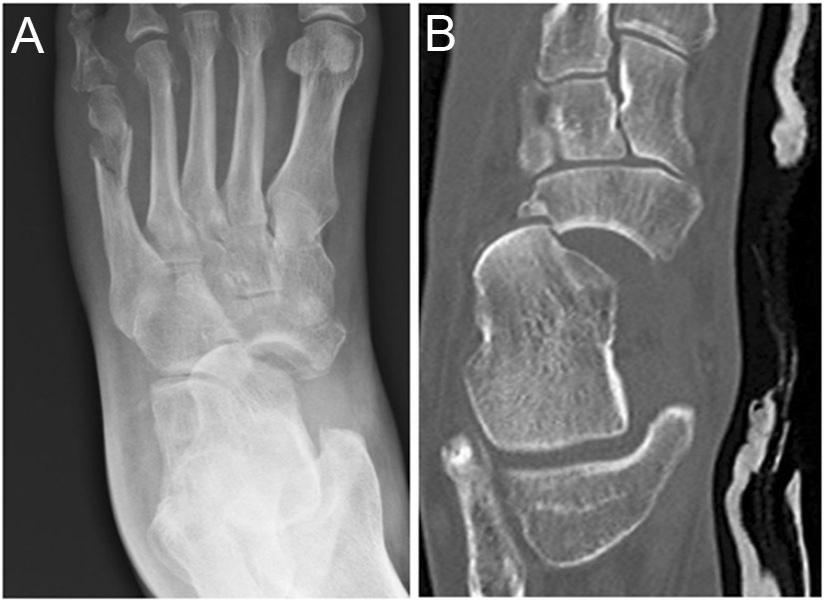
Chopart TNJ fracture-dislocation example. Anteroposterior radiograph (**A**) and axial CT (**B**) of a talar head dislocation with medial talar head and lateral navicular impaction injury

Chopart injuries can be allocated into 4 broad groups, ligamentous injury with or without dislocation and fracture with or without dislocation, which must occur at the TNJ and/or CCJ. Chopart dislocations include both pure-dislocations and fracture-dislocations. Pure-dislocations are defined as dislocation of the navicular and/or cuboid without associated fracture. Fracture-dislocations are defined as dislocation of the navicular and/or cuboid with associated fracture of one or more of talus, navicular, calcaneus or cuboid including avulsion fractures. Dislocation of both the TNJ and CCJ simultaneously, may be referred to as a complete Chopart dislocation. Swivel dislocations typically result from medial or lateral deforming forces causing TNJ and/or CCJ dislocation and the calcaneus ‘swivels’ on an intact talocalcaneal ligament [7]. We have provided radiological examples of a Chopart fracture (Fig. 2) and fracture-dislocation (Fig. 3). We have also provided an explanation of Main and Jowett’s landmark classification [8] of Chopart injuries, with a focus on dislocations (Table 1).

**Table 1:** Explanation of Main and Jowett’s classification [8] of Chopart injuries, according to direction and magnitude of force, with guidance [9, 10]. Talonavicular joint (TNJ), Calcaneocuboid joint (CCJ)

Type	Description
1. Medial Forces	Mild—sprain
	Moderate—Navicular fracture and/or TNJ dislocation
	Severe—Medial swivel dislocation (TNJ ± CCJ) ± Navicular fracture
2. Axial forces	Foot in plantar flexion at impact and longitudinal force transmitted along metatarsal rays causing compression of foot columns
	Mild—sprain
	Moderate—Central Navicular fracture and/or TNJ dislocation
	Severe—Dorsal dislocation (TNJ ± CCJ) commonly with associated midfoot fracture
3. Lateral Forces	Mild-sprain
	Moderate—Cuboid/anterior calcaneal fracture and/or CCJ dislocation
	Severe—Lateral swivel dislocation (CCJ ± TNJ) ± cuboid/anterior calcaneal fracture
4. Plantar Forces	Mild-sprain
	Severe—Fracture-dislocation/pure-dislocation typically of both TNJ and CCJ with associated midfoot fracture
5. Crush Injuries	Random forces of high velocity/energy causing fractures, pure-dislocations and fracture-dislocations

## Aims

We aim to review the literature to provide evidence-based recommendations for the diagnosis and management of Chopart dislocations, to improve outcomes for future patients. We also aim to gain further insight into the incidence and aetiology, prognostic factors and management options for these rare injuries.

## Methods

### Study selection

The electronic databases PubMed, Medline and Scopus were utilised. A preliminary search with no inclusion or exclusion criteria was carried out to gauge the amount of existing literature. This demonstrated a relatively low quantity of existing papers and as a result the set inclusion and exclusion criteria were broad. We included any papers relating to acute traumatic Chopart dislocations and/or Chopart fracture-dislocations in the adult population (Table 2). We excluded papers that were not accessible from the authors online catalogue and any papers not published in English to mitigate translational errors. A literature search was undertaken to identify all related papers. Two search term strings, one anatomical and one related to the type of injury, were combined to narrow down the search to related papers. Applied search terms included “Chopart joint”, “Midtarsal joint”, “Dislocation” and “Fracture-dislocation”. The search identified 181 papers on PubMed/Medline and 280 on Scopus. The PRISMA flow chart was used to scrutinise the initial search results (Table 3). Duplicates were removed and abstracts from these papers were screened for relevance. Articles were selected based on relevance to the topic area and title of the review. The full text of each study was then assessed, and any further non-suitable papers excluded in accordance with the inclusion and exclusion criteria.

**Table 2:** Inclusion and exclusion criteria set for the literature search

Inclusion criteria:
All literature
Papers discussing Chopart dislocation or fracture-dislocation
Papers discussing treatment of Chopart dislocation or fracture-dislocation
Papers on the adult population (≥ 18 years old)
Acute traumatic injuries
Exclusion criteria:
Papers not accessible in the authors online catalogue
Papers not published in English
Hindfoot dislocations
Papers on the paediatric population (< 18 years old)
Non acute, congenital or chronic pathologies

**Table 3:** PRISMA flowchart to scrutinise literature search results

Identification	Database search (*n* = 461)	Additional records from other sources including reference lists (*n* = 15)
Screening	Records after removal of duplicates (*n* = 295)	Excluded by title (*n* = 180)
	Records screened (*n* = 115)	Excluded by abstract (*n* = 53)
Eligibility	Full text assessed (*n* = 62)	Full text articles excluded (*n* = 4)
Included	Total studies reviewed (*n* = 58)	

## Results

We identified 58 papers for review, 36 case reports, 4 cohort studies, 4 case series and 14 other papers related to the epidemiology, diagnosis, treatment and outcomes of Chopart dislocations and fracture-dislocations.

### Cohort studies and case series

### Incidence and aetiology

A retrospective epidemiology cohort study by Ponkilainen et al. of 307 midfoot injuries suggests the incidence of midfoot injury to be 12.1/100,000/year and Chopart injury to be 2.2/100,000/year [16]. Motor vehicle accidents (MVA) are the primary aetiology for Chopart dislocations are more common in males [6, 12]. Richter et al. found 16% of 155 midfoot fractures were Chopart fracture-dislocations [12]. A follow-up study conducted of 110 Chopart-dislocations, 25% were found to be pure-dislocations, 55% fracture-dislocations and 20% combined Chopart-Lisfranc fracture-dislocations [6]. A recent study of 128 Chopart joint injuries found only 5 patients (3.7%) had pure-dislocations and the most frequent fracture-dislocation was transnavicular/transcuboidal in 21% of cases [15]. The average age was 36.8 years (5 studies) (Table 4).

**Table 4:** Cohort Studies and case series identified by literature review on Chopart dislocation and fracture-dislocation functional outcomes, including: 2 retrospective cohort studies, 1 prospective cohort study and 4 case series

Paper	Paper type and aim	Patient number and injury type	Patient characteristics	Diagnosis and radiology	Management	Follow-up group and time	Outcomes
Mittlmeier et al. [11]	Case series—Gait function following Chopart ± lisfranc fracture-dislocation	25 patients: 5 Chopart fracture-dislocations 9 combined Chopart-Lisfranc fracture-dislocations	Not reported	Not reported	ORIF (screws and/or K-wires) ± Ex-fix	Follow up range 1–8 years	Patients tended to load on the non-injured column Severity of post-traumatic arthritis did not greatly influence gait function Loss of either foot column length had a substantial influence in gait quality
Richter et al. [12]	Retrospective cohort study—Midfoot injury outcomes	155 midfoot injuries: 25 Chopart fracture-dislocations 26 combined Chopart-Lisfranc fracture dislocations	Average age—35 years 114 males and 41 females. (2.78:1 M:F) Principal aetiology—MVA (112/155)	Not reported	148/155 (95%)—Operatively 115/155 (74.2%)—OR 30/155 (19.4%)—CR 116/155 (74.8%)—Internal fixation typically with K-wires ± screws 55/155 (35.5%)—additional Ex-fix 3/155 (1.9%)—Primary Below knee amputation (all Chopart-Lisfranc fracture-dislocations (3/26, 11.5%)) 18/155 (11.6%)—Required foot compartment fasciotomy	92 patients—average follow up 9 years	Mean AOFAS-midfoot for Chopart fracture-dislocations—67 (*n* = 15) Mean AOFAS-midfoot for Chopart-Lisfranc fracture-dislocation—55 (*n* = 14) Mean AOFAS-midfoot for Isolated Chopart fracture—81 (*n* = 18) High correlation between correct column length and good functional outcome Early ORIF provided the highest scores in all groups
Richter et al. [6]	Retrospective cohort study—Chopart dislocation outcomes	110 Chopart dislocations: 28 pure-dislocations 60 Chopart fracture-dislocations 22 combined Chopart-Lisfranc fracture-dislocations	Average age—32 years 68 men and 32 women (2.13:1 M:F) Principal Aetiology was MVA (90/110)	Not reported Author recommendation: XR + CT	Pure-dislocations—19/28 CR ± internal fixation (6/19) ± Ex-fix (1/19) Chopart fracture-dislocations—ORIF (50/60) ± Ex-fix (14/60). Primary amputation (3/60) Combined Chopart-Lisfranc dislocations—primary amputation (9/22), ORIF (11/22) ± Ex-fix (5/22) Average time between injury and operative treatment—3 days 28/110 (25.5%)—required foot compartment fasciotomy	58 patient with 59 Chopart dislocations: average follow-up 9 years	Mean AOFAS whole group 75 (*n* = 59) Mean AOFAS Pure-dislocation—79.0 (*n* = 14) Mean AOFAS Chopart fracture-dislocation—78.0 (*n* = 33) Mean AOFAS Chopart Lisfranc fracture-dislocation—61 (*n* = 12) No significant differences for age or gender Negative prognostic factors: MVA, Open injury, associated fractures, polytrauma, combined Chopart lisfranc injuries, delayed surgery > 1 day Positive prognostic factors: closed or isolated injuries, compartment syndrome Open reduction prior to internal fixation had significantly better outcomes than closed reduction prior to internal fixation
Rammelt et al. [13]	Case series—secondary reconstruction of malunited Chopart fracture-dislocations	8 patients with malunited Chopart fracture-dislocations	Average age: 38 years Surgical Revision: average 10 months post injury	Not reported	ORIF—7/8 TNJ fusion—1/8	8 patients average follow-up of 2 years	Mean post-operative AOFAS—80.8 (*n* = 6) Mean pre-operative AOFAS—38.8 (*n* = 6) Highly significant difference Active inversion/eversion: averaged 18 degrees preoperatively and 41 degrees postoperatively
Van Dorp et al. [5]	Case series—Chopart dislocation outcomes	9 patients: 7 fracture-dislocations, 2 Pure-dislocations	Average age: 41.6 years 2:1 M:F Sprain/sport injury: 5 MVA: 3 Fall from height:1	XR + CT 2 patients the injury was initially 3 patients with underestimated injury prior to CT	Average time between injury and surgery—7 days 5 patients non-operative 1 patient CREF 3 ORIF (2 + Ex-fix) Complications of surgery: 1 Ex-fix pin track infection 1 persistent dislocation of TNJ 1 secondary dislocation 5 days post-op requiring further surgery	7 patients average follow-up—31.3 months	Mean AOFAS—72 (*n* = 7) Mean VAS patient satisfaction score—7.1/10 (*n* = 7) 4/7—pain free 1/7—moderate pain 2/7—daily pain 4/7—stiffness 5/7—returned to work, 1 unable and 1 already retired 5/7—limitations performing sports or leisure
Kosters et al. [14]	Case series—Chopart fracture-dislocation	24 patient cohort with 6 fracture-dislocations	Mean BMI: 23.0 Median Age: 42 years	Not reported	ORIF/CREF	Median follow up: 2.6 years	Mean AOFAS—66.0 (*n* = 6) Maryland foot score—80.8 (*n* = 6) SF-36 (quality of life score)—61.4 (*n* = 6)
Rammelt et al. 2023 [15]	Prospective cohort study—Chopart injury outcomes	128 Chopart joint injuries: 5 (4%) pure-dislocations 21.1%—transnavicular/transcuboidal fracture-dislocations 23.4% Combined Chopart-Lisfranc fracture dislocation	Average age—37.3 years 83 male, 39 female patients. (2.13:1 M:F) MVA—45.9% Direct trauma to foot—24.6% Fall from height—26.2% Low energy trauma—4%	XR + CT Diagnosis delayed for > 24 h in 27 patients (22.1%)	11/128 (8.6%)—non-operatively 87/128 (68%)—Single stage surgery 30/128 (23.4%)—Staged treatment 19/128 (14.8%)—Pure-dislocations/fracture–dislocations had OR/CR followed by K-wire fixation ± Ex-fix 12/128 (9.4%)—Compartment syndrome (immediate release) 33/128 (25.8%)—required bone grafts 7/128 (5.5%)—required primary arthrodesis	73 patients with 75 Chopart injuries average follow-up: 10.2 years	Mean AOFAS for Chopart Injuries (fractures, pure-dislocations, fracture-dislocations)—71.5 (*n* = 75) Negative prognostic factors: High injury severity score, work-related accidents, open and multiple fractures, pure-dislocations, staged surgery, delay of treatment > 4 weeks, post-operative infection and primary/secondary fusion ORIF provided significantly better outcomes than closed reduction and percutaneous fixation Pure-dislocations had worst prognosis

Open Reduction (OR), Closed Reduction (CR), Medial Column Length (MCL), Lateral Column Length (LCL), Open Reduction and Internal Fixation (ORIF), External Fixation (Ex-fix), Talonavicular joint (TNJ), American Orthopaedic Foot and Ankle Society Score (AOFAS), Motor Vehicle Accident (MVA). Number of patients (n), Male to female ratio (M:F).

### Diagnosis

5/7 studies did not report method of diagnosis. Of the two studies that did, XR plus CT scanning was used. One reported delayed diagnosis for more than 24 h in 22.1% of the 122-patient cohort [15]. In the second, two injuries were initially missed after inspection of plain films and injury severity was underestimated in three injuries prior to CT scanning (Table 4) [5].

### Management

83% of 60 Fracture-dislocations were managed with ORIF ± external fixation [6]. 19 of 28 pure-dislocations were managed initially with closed reduction, although six of these required additional internal fixation and one external fixation [6]. Compartment syndrome requiring fasciotomy was reported in 11.6% of 155 midfoot injuries [12], 9.4% of 128 Chopart injuries [15] and 25.5% of 110 Chopart dislocations [12]. Zero percent of pure-dislocations, 5% of fracture-dislocations and 25% (2 studies, *n* = 48) of combined Chopart-lisfranc fracture-dislocations required primary amputation [6, 12]. 7/128 Chopart injuries required primary arthrodesis and 4.7% of cases required late fusion at the Chopart joint [15].

### Outcomes

Average AOFAS score for each Chopart injury classification across the cohorts and case series reviewed (Table 4). Isolated Chopart fractures: 81.0 (1 study, *n* = 18). Chopart fracture-dislocations: 70.3 (3 studies, *n* = 54). Chopart pure-dislocations: 79.0 (1 study, *n* = 14). Combined Chopart-Lisfranc fracture dislocations: 58.0 (2 studies, *n* = 26).

Richter et al. reported Isolated Chopart fractures had significantly better AOFAS-Midfoot scores than Chopart fracture-dislocations [12]. The highest scores in all groups were achieved in those fractures treated with early ORIF. No significant differences in the scores were found for age, gender or aetiology. Radiographic comparison of Chopart fracture-dislocation (*n* = 15) against isolated Chopart fracture (*n* = 18) at an average follow up of 9 years. Incorrect medial column length 27% vs 6%. Incorrect lateral column length 20% vs 11%. Abnormal longitudinal arch shape 40% vs 22%. Arthritic changes 67% vs 33% [12]. There was a high correlation correct column length and good functional outcomes [12]. Furthermore, Mittlmeier et al. found the loss of foot column length had a substantial influence on gait quality and the severity of post-traumatic arthritis had no significant influence [11].

In a follow up study by Richter et al. focussed on Chopart dislocations, those who underwent internal fixation following closed reduction had significantly worse outcomes than those who underwent ORIF in the first place [6]. No significant differences in functional outcomes were found between age, gender or methods of internal fixation when comparing screws and/or K-wires [6]. AOFAS scores were significantly lower in MVA than in non-MVA and in open injuries versus closed. Polytrauma, associated fractures and delayed surgery > 1 day from injury were also significant negative prognostic factors. Those with compartment syndrome had significantly better outcomes, however these patients all had expedited surgery within 24 h [6].

Rammelt et al. followed up 75 Chopart injuries for an average of 10.2 years [15]. ORIF led to significantly better results that closed reduction and percutaneous fixation across all Chopart injuries including pure-dislocations and fracture-dislocations [15]. Only 4% of 128, Chopart injuries were pure-dislocations which had significantly inferior Foot Function Index and AOFAS scores than patients with Chopart fractures or fracture–dislocations [15]. Negative prognostic factors included, high injury severity score, work-related accidents, open and multiple fractures, pure-dislocations, staged surgery, delay of treatment > 4 weeks, post-operative infection and primary or secondary fusion [15].

Van Dorp et al. presented 7 patients with Chopart dislocations [5]. The mean AOFAS score was 72 and the mean VAS score for patient satisfaction was 7.1/10 at an average 31.3 month follow-up [5]. 4/7 patients still experienced pain or limitation of daily activities at follow-up [5]. Another case series by Kosters et al. saw 6 patients with Chopart fracture-dislocations report a mean AOFAS of 66, half of patients were found to have post-traumatic arthritis and one patient had pathological medial column length, at a median follow-up of 2.6 years [14]. Eight malunited Chopart fracture-dislocations underwent secondary anatomic reconstruction, on average 10 months following injury [13]. Seven patients had joint sparing reconstruction with ORIF, and one TNJ arthrodesis. The mean AOFAS pre-operatively and at 2-year follow-up were 38.8 and 80.8 respectively, (*p* < 0.0001). Active inversion/eversion of the foot (total coronal plane motion) averaged 18° preoperatively and 41° at follow-up, excluding the TNJ fusion [13].

### Case reports—Chopart pure-dislocation

Twelve case reports of pure-dislocations were identified for review (Appendix Table 5). The average age was 39.1 with a 3:1 male to female ratio. Half of injuries were caused by MVA and One third were precipitated by falls. 5/12 were complete Chopart dislocations. 5/12 reports were isolated swivel dislocations of the navicular (4 medial, 1 lateral), which was most frequently dislocated in isolation (7/12). The CCJ was not found to dislocate in isolation. The plantar ecchymosis sign was not reported in any cases. Imaging most frequently consisted of XR plus CT (7/12). In nine cases, closed reduction was attempted which failed in four. Of the five cases treated with closed reduction, three were managed non-operatively. Seven out of twelve cases underwent open reduction with additional internal fixation of five and arthrodesis of two. Follow up was provided for 10 cases between six and 76 months. Five of these had significant long-term complications, which ranged from pain on prolonged standing, to pes planus and midfoot arthritis.

### Case reports—Chopart fracture-dislocation

24 case reports of Chopart fracture-dislocations were identified (Appendix Table 6). The average age was 34.8 with a 2.4:1 male to female ratio. The most common aetiology was MVA in 10 reports. According to dislocation type, 13 were TNJ dislocations, 6 CCJ dislocations and 5 complete Chopart dislocations. The calcaneus was most commonly fractured (13/24), followed by the navicular (10/24), cuboid (7/24) and talus (4/24). Eleven reports used XR alone for diagnosis, 13 used additional CT. Two Chopart fracture-dislocations were initially missed on XR. The plantar ecchymosis sign was reported in two cases. Where closed reduction was attempted, 7/18 failed. 11 cases were successfully reduced, although 8 required surgical fixation. Thirteen cases had open reduction, definitive management was ORIF for 11 and arthrodesis for 2. Follow up ranged from 3.5 months to 5 years with 2 cases lost to follow-up. 13/22 cases had sequalae, ranging from pain to pes planus and malunion. 3/22 of cases required further surgery. Eight cases had definitive treatment more than 48 h after injury, six of these had complications at follow-up. Seven out of twenty-four cases provided AOFAS scores between 3.5 and 24 months, with a mean score of 87.7.

## Discussion

### Clinical signs

The plantar ecchymosis sign (PES) describes a central midfoot plantar ecchymosis that is pathognomic for relevant midfoot injuries indicating rupture of strong plantar ligaments and resulting haematoma [17]. PES has been recorded following calcaneal fractures [18], Lisfranc [19] and Chopart injuries [20]. PES was not reported in the cohort studies or case series reviewed, although 2/36 case reports reported the PES. The PES is a rarely reported but valuable clinical sign indicating underlying midfoot injury, CT is encouraged where PES is positive and radiographs negative. Clinicians should carefully examine and palpate the entire foot alongside confirming the neurovascular status and have a high index of suspicion for compartment syndrome which is common in Chopart injuries [12, 15], especially dislocations [6].

### Imaging

The wider literature has shown, up to 41% of Chopart injuries are missed at first presentation [5]. Haapamaki et al. found plain radiographs alone missed 33% of fractures in Chopart injuries [21]. From Van Dorp et al. Case series of 9 Chopart dislocations, 2 were initially missed from XR alone and the severity of injury was underestimated for 3 prior to CT, alongside this 2/24 fracture-dislocations from case reports were initially missed on XR (Appendix Table 6). Furthermore Rammelt et al. found pure-dislocations to be very rare (4%) and encourage CT scanning to rule out associated fracture [15]. This is in concordance with Almeida et al. who found a significant improvement in identifying additional Chopart fractures missed on XR with CT [22]. Furthermore, CT allows reconstructive modelling to determine the degree of dislocation [4]. We recommend CT to be included in the diagnostic workup of all suspected Chopart injuries.

### Management and outcomes

For Chopart dislocations including pure-dislocations and fracture-dislocations, initial ORIF provides better outcomes than closed reduction prior to internal fixation [6]. Closed reduction alone was found to have statistically similar outcomes to operative treatment [6], however, 6 of 14 pure-dislocations required internal fixation following closed reduction and therefore should have undergone ORIF initially. Due to the risk of requiring internal fixation following closed reduction, urgent ORIF is advised and closed reduction should be discouraged. Furthermore, closed reduction is challenging, often fails and repeated attempts may cause further damage [23]. For Chopart injuries in general including isolated fractures, fracture-dislocations and pure-dislocations, Rammelt et al. found over a 10-year follow up that ORIF generates significantly better results than closed reduction and percutaneous fixation [15]. It is important to note that according to Rammelt et al. [15] pure-dislocations had the worst prognosis, however, AOFAS scores were not provided and could not be included in the average across studies.

Maintenance of foot column length significantly improves gait quality [11]. We agree with Van Dorp et al. that correct alignment of the foot axes and correct length of medial and lateral columns should be a major goal of therapy [5], these have been regularly incorrect at follow up, thus this should be addressed peri-operatively and corrected where possible. For malunited Chopart fracture-dislocations, secondary reconstruction improves outcomes in suitable patients otherwise joint fusion is required [13]. Arthrodesis is implemented in late presentations [24], in patients with diabetic arthropathy [25], or when other treatment strategies have failed where arthrodesis can prevent midfoot collapse [26]. TNJ Arthrodesis can reduce the Chopart joint range of motion by 50% and is a negative prognostic factor [15], so is considered a last resort [27]. Soft tissue condition can help to dictate treatment methods, where external fixation can maintain reduction during soft tissue healing [15, 28] and maintain column length with unstable ORIF [29]. Patients with Chopart pure-dislocations and fracture-dislocations require long term follow-up to monitor for complications which were common [5, 14].

### Limitations

There was marked heterogeneity of the reviewed studies in terms of injury pattern, injury classification, treatment modality and outcome reporting which led to difficulties in performing systematic review, which was our original intention. This was coupled with the paucity of research on this topic due to the rarity of the injury itself.

## Conclusion

This review focussed on Chopart dislocations. Pure-dislocations appear to have inferior outcomes due to the high energy required to disrupt the strong ligamentous anatomy at the Chopart joint. There is a general consensus that closed reduction often fails and leads to poorer outcomes, even if followed by ORIF. Besides joint reconstruction, restoring and maintaining the medial and lateral foot columns is essential to obtain reasonable results. CT and compartment syndrome evaluation is highly recommended. Urgent ORIF ± external fixation is the management of choice for pure-dislocations and fracture-dislocations. Negative prognostic markers included, severity of injury, delayed or staged treatment, arthrodesis and MVA.

## Appendix

See Tables 5 and 6.

**Table 5:** Chopart pure-dislocation case reports derived from the literature search

Study (*n* = 12)	Age	Sex	Mechanism of injury	Dislocation type	Imaging	Treatment	Immobilisation and hardware removal	Follow up
Jung et al. [30]	78	F	Inversion whilst descending stairs	Medial swivel TNJ dislocation	XR + CT + MRI	Failed closed reduction ORIF (K wires)	6w of non-WBBK cast K-wires removed at 12w, partial WB for 2w and full WB by 14w	1 year—no Complications AOFAS—85
Honeycutt et al. [23]	48	M	Fall 3-foot, landing in plantar flexion	Plantar Chopart dislocation	XR + CT	Failed Closed reduction ORIF (plate + screws)	7w of non-WBBK splint, 4 more weeks of non-WB walking boot. Full WB from 12w	20-months—fatigue after prolonged standing, unable to run
Kumar et al. [24]	48	F	Fall (2 months ago)	Medial swivel TNJ dislocation	XR + CT	Closed reduction not attempted. Open reduction + arthrodesis	6w of non-WBBK splint followed by progressive WB	6 months—no complications
Datt et al. [31]	35	M	MVA (6 weeks ago)	Medial swivel TNJ dislocation	XR + CT	Closed reduction failed ORIF (K wires)	6w of non-WBBK cast. K-wires removed at 6w followed by progressive WB	32 months—no complications
Genena et al. [32]	30	M	MVA	Dorsal Chopart + NC dislocation	XR + CT	Failed closed reduction ORIF (K wires)	6w of non-WBBK cast followed by progressive WB. K-wires removed at 4w	12 months—Midfoot pain, pes planus, midfoot arthritis
Ansari et al. [26]	64	M	MVA	Complete dislocation of Navicular	XR + CT	Closed reduction was not attempted, TN arthrodesis	12w of non-WB posterior slab Followed by progressive WB	Lost to follow-up
Puthezhath et al. [33]	35	F	Fall from 6-foot landing in plantar-flexion	Dorsal dislocation of the TNJ	XR	Closed reduction not attempted ORIF (K wires)	6w of non-WBBK cast, K-wires removed at 12w followed by Progressive WB	96 weeks—midfoot pain on prolonged standing
Ip et al. [34]	45	M	Fell from 4 m	Dorsal Chopart dislocation	XR	Closed reduction and internal fixation with screws	6w of non-WBBK cast, screws removed at 20w	76 month—Midfoot pain on prolonged walking or standing
Harris et al. [35]	24	M	Crush injury by construction vehicle (MVA)	Plantar Chopart dislocation + intercuneiform dislocation	XR + CT	Closed reduction	6w of non-WBBK cast Followed by tolerated WB in CAM boot until 12w	12 weeks—dorsal ossification of Chopart joint + TNJ degenerative change
Inal et al. [36]	18	M	MVA	Medial swivel TNJ dislocation	XR + CT	Closed reduction	6w of non-WBBK cast Patient returned to normal daily activities following this	14 months—no complications
Ruthman et al. [37]	19	M	Crush injury by construction equipment	Plantar Chopart dislocation	XR	Closed reduction	Non-WBBK cast (no time frame provided)	Lost to follow-up
Kapila et al. [38]	25	M	MVA	Lateral swivel TNJ dislocation	XR	Closed reduction + K wire fixation	6w of non-WBBK cast with K-wire removal at 6w. 8w tolerated WB, full WB at 12 weeks	1 year—no complications

This table includes 12 case reports of Chopart pure-dislocations. Cases with associated fractures outside of the Chopart joint were permissible to this category. Times have been provided in the mechanism of injury column in the case of late presentation. Any injury involving vehicles were classed as motor vehicle accidents (MVA). Talonavicular joint (TNJ). Calcaneocuboid joint (CCJ). Naviculocuneiform joints (NCJ). Below-Knee (BK). Weight-bearing (WB). Weight-bearing below-knee (WBBK). Six weeks (6w). American Orthopaedic Foot and Ankle Society Score (AOFAS). Number of patients (*n*)

**Table 6:** Chopart fracture-dislocation case reports derived from the literature search

Study (*n* = 24)	Age	Sex	Mechanism of injury	PES	Fracture-dislocation type	Imaging	Definitive treatment	Immobilisation and hardware removal	Follow up
Dhole et al. [39]	30	F	Fall from 2-foot	−ve	CCJ + NCJ dislocation + AP calcaneus fracture	XR	Closed reduction failed, ORIF (K-wires)	8w of non-WBBK cast followed by K-wire removal and WB as tolerated	24 months: AOFAS 95, mild pain whilst fast walking and running
Donthi et al. [40]	27	M	Fall from 7-foot, 3 days prior	−ve	Fracture-dislocation of navicular + cuboid fracture	XR	Closed reduction + K wire fixation	12w of non-WBBK cast followed by K-wire removal and progressive WB	1 year: asymptomatic non-union of the navicular and cuboid, 2-years—No complications
Kanwat et al. [41]	20	F	Fall from 10-foot, 7 weeks prior	−ve	Medial swivel TNJ dislocation + calcaneus and cuboid fracture	XR + CT	Closed reduction was not attempted ORIF (k-wires + Ex-Fix)	4w Ex-fix removed 6w K-wires removed with progressive WB. Full WB at 12w	1 year: no complications
Gonzales et al. [42]	19	F	Struck dorsal foot in plantar flexion 8 weeks prior	−ve	Plantar Chopart dislocation + AP calcaneus and TH fracture	XR + CT Initially missed by XR	Closed reduction was not attempted ORIF (K-wires)	6w of non-WBBK cast. hardware and cast removed at 6w followed by partial WB	12 months: AOFAS 74, functional limitation when running
Layson et al. [43]	32	M	Fall from second storey balcony	−ve	Medial swivel TNJ dislocation + navicular, cuboid and AP calcaneus fracture	XR + CT Initially missed by XR	Closed reduction failed ORIF (K-wires + Plate)	12w of non-WBBK splint. Hardware removed at 12w	5 years: no complications
Hagino et al. [44]	42	M	MVA	−ve	CCJ dislocation + AP calcaneus fracture	XR + CT	Closed reduction was not attempted ORIF (K-wires)	3w non-WBBK splint. K-wires removed at 8w followed by progressive WB	2 years 6 months: JSSF 90, midfoot pain on prolonged walking or standing
Davis et al. [45]	29	M	MVA	−ve	Dislocation of TNJ with calcaneal and cuboid fractures	XR	Closed reduction, K-wire fixation + Ex-Fix	7w of non-WB when K-wire and Ex-Fix removed 7w onwards non-WBBK boot	23 weeks: mild rest pain and swelling
Alayed et al. [46]	27	M	MVA	−ve	CCJ + NCJ dislocation with calcaneus fracture	XR	Closed reduction failed ORIF (K wires)	12w of non-WBBK splint. K-wires removed at 12w followed by progressive WB	12 months: no complications, AOFAS 90
Rao et al. [47]	85	F	MVA	−ve	Open complete dislocation of navicular + calcaneus fracture	XR	Closed reduction not attempted, open reduction + arthrodesis of NCJ and CCJ + K wires	8w of non-WBBK posterior splint. K wires removed at 4w + partial WB Full WB at 16w	24 months: no complications
Samoladas et al. [48]	24	M	Fall playing football	−ve	Dislocation of the TNJ + navicular fracture	XR	Closed reduction (Post-reduction XR found TH impression fracture)	4w non-WBBK cast	2 years: heterotopic ossification of dorsal TNJ due to TH fracture, early indication of arthritis
Johnstone et al. [28]	20	M	MVA (15 days prior)	−ve	Fracture-dislocation of navicular	XR	Closed reduction not attempted. Open reduction, TNJ arthrodesis + K-wires, NCJ K-wires	6w of non-WBBK cast. Followed by 6w partial-WB Cast and K-wires were removed at 12w and full WB at 18w	24 months: Running discomfort or walking on uneven ground. Reduction of Chopart joint ROM by approx. 50%
Schildhauer et al. [49]	27	M	Fall from 5 m	−ve	Complete lateral Chopart dislocation + navicular and cuboid fracture from crush	XR	Day 7—Internal fixation (bridge plate for navicular and plate for cuboid) following closed reduction and Ex-fix for soft tissue recovery	12w of toe-touch WB 6w K-wires removed and medial column bridge plate was shortened to allow for restoration of TNJ motion	One year: all hardware was removed because of soft tissue irritation at the dorsum of his foot when wearing shoes
Harris et al. [50]	25	M	Missed step descending stairs	−ve	Dorsal Chopart dislocation + navicular fracture	XR + CT	Closed reduction failed ORIF	Non-WB (no timeframe specified)	Lost to follow up
Fares et al. [51]	20	M	Forklift Crush injury (MVA)	PES + ve	CCJ + NCJ dislocation + cuboid impaction fracture and avulsion fracture of anterior calcaneal process	XR + CT	Closed reduction failed ORIF (CCJ K-wires, NC Plate)	8w of BK cast, K wires removed at 12w	1 year: patient complains of stiffness and intermittent pain on walking removal of hardware is planned
Alhadhoud et al. [52]	25	M	Forklift crush injury (MVA)	−ve	Open fracture dislocation of CCJ + NCJ + AP calcaneal fracture	XR + CT	Internal fixation 5 weeks following closed reduction due to infection NC1/2 plate, CCJ K-wire	5w non-WBBK cast during infection Non-WB no cast for 6 weeks after ORIF followed by progressive WB	14 weeks: VAS 0/10, AOFAS 83, EQ5D 90, no complications
Puente et al. [53]	65	F	Unknown	−ve	Plantar dislocation of navicular + cuboid fracture	XR	Closed reduction not attempted. ORIF (K wires)	6w of non-WBBK cast. K wires removed at 6w with progressive WB	2 years: no complications
Algouaiz et al. [54]	25	M	Twisting	−ve	Medial TNJ swivel dislocation + Navicular fracture	XR + CT	Closed reduction	6w of non-WBBK cast	6 months: Unable to run due to pain
Mathesul et al. [55]	50	M	MVA—plantar flexion with axial load	−ve	Fracture dislocation of the navicular	XR + CT	Closed reduction + Ex fix and K wires	6w of non-WB Ex-fix and K wires removed at 6w + partial WB, full WB at 12w	12 months: no complications, AOFAS 97
Pillai et al. [7]	45	F	Fall from 3 feet	−ve	Lateral swivel TNJ dislocation with cuboid compression fracture	XR	Closed reduction + K wire fixation	4w of non-WBBK cast. Cast and K-wires removed at 4w + partial WB and full WB at 10w	24 months: no complications, AOFAS 88
Williams et al. [56]	22	F	Inversion when walking	−ve	Medial swivel TNJ dislocation with cuboid fracture and talar avulsion fracture	XR	Closed reduction alone	3w of non-WBBK cast followed by progressive WB Full WB at 9w	18 months: intermittent mild discomfort and reduced ability to dance. AOFAS 87
Powell et al. [57]	46	M	Fall landing flexed and inverted	−ve	Medial swivel TNJ dislocation with cuboid, navicular and talar fractures	XR + CT	Closed reduction + K wire fixation	8w of WB in CAM boot. K-wires removed at 6w. 11w Full WB	Lost to follow up
Kummer et al. [58]	34	M	Crush to posterior foot by construction vehicle (MVA)	−ve	CCJ + NCJ Dislocation + anterior calcaneal avulsion fracture	XR + CT	Failed closed reduction and percutaneous fixation, ORIF 2 weeks later	6w of partial-WBBK cast. K-wires removed at 6w + progressive WB	1 year: needs surgery to remove osseous fragment + Hardware
Schmitt et al. [59]	35	M	Fall from 1.5 m	PES + ve	Dorsal Chopart dislocation + Talar avulsion fracture	XR + CT	Closed reduction + Ex-Fix	Ex-fix removed at 4w Non-WBBK cast for 4w, followed by tolerated WB	6 months: no complications
Viegas et al. [60]	62	M	MVA	−ve	Plantar Chopart Dislocation + calcaneus and navicular fracture	XR + CT	Closed reduction failed—several days later ORIF + pins	Non-WBBK cast to partial-WB over 6 weeks. Cast and pins removed at 6w	16 months: Pain + pes planus, plantar dislocation and arthritis, requiring triple arthrodesis

This table includes 24 case reports of Chopart fracture-dislocations. Times have been provided in the mechanism of injury column in the case of late presentation. Any injury aetiology involving vehicles were classed as motor vehicle accidents (MVA). Cases with associated additional fractures outside of the Chopart joint were permissible to this category. Plantar Ecchymosis Sign (PES). Japanese Society for Surgery of the Foot (JSSF). American Orthopaedic Foot and Ankle Society Score (AOFAS). Visual Analogue Scale (VAS). EuroQuol 5-dimension (EQ5D). Talonavicular joint (TNJ). Calcaneocuboid joint (CCJ). Naviculocuneiform joints (NCJ). Talar head (TH). Below-Knee (BK). Weight-bearing (WB). Weight-bearing below-knee (WBBK). 6 weeks (6w). Number of patients (*n*)
